# COVID-19 and Antipsychotic Therapy: Unraveling the Thrombosis Risk

**DOI:** 10.3390/ijms25020818

**Published:** 2024-01-09

**Authors:** Eszter-Anna Dho-Nagy, Attila Brassai, Patrick Lechsner, Corina Ureche, Erika-Gyöngyi Bán

**Affiliations:** 1 Department of Pharmacology and Clinical Pharmacology, Faculty of Medicine in English, Preclinical Research Laboratory, George Emil Palade University of Medicine, Pharmacy, Science, and Technology of Targu Mures, 540142 Targu Mures, Romania; 2 Department of Internal Medicine, Faculty of Medicine, George Emil Palade University of Medicine, Pharmacy, Science, and Technology of Targu Mures, 540142 Targu Mures, Romania

**Keywords:** antipsychotic medications, thrombosis, COVID-19

## Abstract

In the context of the COVID-19 pandemic, this study investigates the potential correlation between the increased use of antipsychotic medications and the rising incidence of venous thromboembolism (VTE). As psychiatric disorders surged, the consequential escalation in antipsychotic drug use raised concerns about thrombotic risks. We conducted a comprehensive literature review using PubMed, focusing on articles that intersected COVID-19, antipsychotic medication, and thrombosis. This approach allowed for a nuanced examination of the historical and recent data on antipsychotic drugs and their association with thrombotic events. Our findings reveal a notable link between the use of antipsychotic medications, particularly second-generation antipsychotics, and an increased risk of VTE, including pulmonary embolism and deep vein thrombosis. This association was evident, despite variations in study designs and populations. The study underscores the need for cautious medication management in psychiatric care, especially during pandemic conditions like COVID-19, to mitigate thrombotic risks. It advocates a personalized approach to prescribing antipsychotics, considering individual patient factors and comorbidities, to balance the benefits against potential thrombotic complications.

## 1. Introduction

In the shadow of the COVID-19 pandemic, a secondary crisis has emerged, marked by a significant rise in psychiatric disorders [1,2]. This increase has necessitated the escalated use of antipsychotic medications, a response that brings its own set of complexities and concerns. Concurrently, there has been a noticeable uptick in the incidence of venous thromboembolism (VTE), a condition characterized by the formation of blood clots in the venous system [1].

The parallel surge in both psychiatric disorders and VTE cases during this period has prompted a compelling question: is there a link between the increased use of antipsychotic medications and the heightened risk of venous thromboembolism?

Our research embarks on this quest, aiming to unravel the potential connections between the management of psychiatric illnesses with antipsychotic drugs and the occurrence of VTE. This exploration is not merely academic; it holds significant implications for public health, offering insights into managing the dual challenge of psychiatric care and thrombotic risks in the context of a global pandemic.

## 2. Materials and Methods

### 2.1. Literature Search Strategy

Our research commenced with an extensive literature review utilizing PubMed as our primary database. The initial search strategy was designed to explore the intersection between COVID-19, antipsychotic medication, and thrombosis. We employed a combination of the three keywords “COVID”, “antipsychotic”, and “thrombosis” to ensure a comprehensive capture of relevant studies. This initial query resulted in the identification of only four articles, indicating a scarcity of research directly addressing all three aspects simultaneously.

To broaden our research scope while maintaining relevance, we adjusted our search strategy to examine the links between keywords. A search combining “antipsychotics” and “thrombosis” was conducted, yielding a more substantial pool of 312 articles. This indicated a well-explored link between antipsychotic medication and thrombotic events in the existing literature.

In our review, we excluded studies reporting thromboembolic events in patients using antipsychotics who also suffered from end-stage diseases; these were omitted due to the inherently higher thrombotic risk in these populations, which could have confounded our assessment [3,4,5]. Additionally, we excluded studies focusing on postoperative patients, as the increased baseline risk for thromboembolism associated with surgery could potentially skew our analysis of antipsychotic-related risks [6,7,8].

### 2.2. Article Selection and Analysis

The selection process involved a preliminary screening of titles and abstracts to identify studies that directly addressed the research question. Full-text articles were then evaluated in depth. The inclusion criteria were centered on studies that provided empirical data or comprehensive reviews related to our key themes: the impact of COVID-19 on thrombotic risks and the role of antipsychotic medications in this context.

Our exclusion criteria encompassed studies involving antipsychotic use in patients with end-stage diseases, as well as those on postoperative patients, where thromboembolism risk could have biased our analysis.

Our analysis involved synthesizing findings from the selected publications, with a particular focus on identifying commonalities and discrepancies in the reported results. This process allowed us to construct a nuanced understanding of the current state of knowledge regarding the links between COVID-19, antipsychotic medication use, and thrombosis.

### 2.3. Inclusion of a Research Methodology Flow Chart

**Figure 1:**
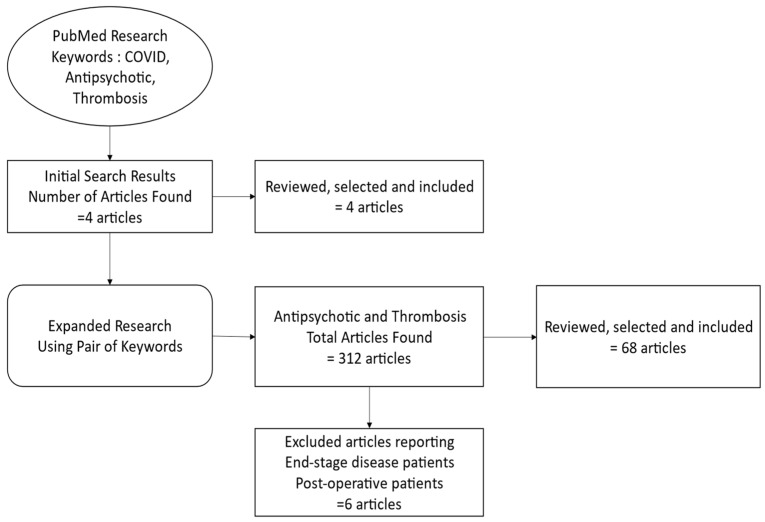
Flow chart outlining the literature search and article selection process.

To enhance the clarity and transparency of our research methodology, we included a detailed flow chart that outlines our literature search and article selection process. This flow chart visually represents the step-by-step approach we employed, from the initial broad search to the final selection of relevant articles (Figure 1).

## 3. Results

### 3.1. Historical Approach: Antipsychotic Medications and Venous Thromboembolic Risk

The historical literature on antipsychotic drugs reveals a longstanding awareness of their potential link to venous thromboembolism, particularly pulmonary embolism (PE). The discovery of chlorpromazine’s antipsychotic properties in 1953 was quickly followed by German case reports citing fatal pulmonary embolism linked to its use, as noted by Brehmer & Ruckdeschel (1953) and Labhardt (1954) [9]. An early case series covering 1954–1957 reported venous thrombosis and pulmonary embolism in 3.3% of phenothiazine users, a notable contrast to a non-phenothiazine control group (Grahmann & Suchenwirth, 1959) [9,10].

Further studies continued to highlight this risk. In 1963, Mahmodian observed a threefold increase in thrombosis risk from 1958 to 1961 in psychiatric and neurological patients compared to earlier decades. Lal et al. (1966) reported a 10% prevalence of PE in autopsies of psychiatric patients, primarily diagnosed with schizophrenia and chronic brain syndrome, matching the general hospital population’s prevalence, suggesting a high incidence of PE in these patients [11].

Kendel and Fodor (1969) found a 29% incidence of PE in psychiatric patients, with a notable association with acute psychiatric symptoms [10]. Scholz (1967) studied psychiatric patients with PE as the clinical cause of death, finding that many had used antipsychotic drugs without any explanatory comorbidity [10]. Ziegler (1977) noted that 4% of autopsy reports with PE as the sole cause of death had an underlying psychiatric disorder [10].

In a large observational study, Meier-Ewert et al. (1967) compared patients with schizophrenia or depression taking chlorpromazine, amitriptyline, or imipramine to similar patients not using these medications [12]. They observed a higher frequency of thromboembolic complications in the medication group (2.9% versus 0.59%) [12]. A study in 1984 reported deep venous thrombosis leading to fatal pulmonary embolism in women with schizophrenia after an acute psychotic phase, highlighting the risk in psychiatric patients [13].

Although there was a gap in the literature post-1984, the topic regained attention with studies by Walker et al. (1997) and Hagg et al. (2000), reflecting continued interest in and concern about the association between antipsychotic medication and venous thrombosis in psychiatric patients [14].

Various antipsychotic drugs, particularly phenothiazines, are known to increase platelet aggregation. However, clinical reports detailing deep vein thrombosis and other thromboembolic phenomena associated with antipsychotics are scarce. Dally noted that chlorpromazine might induce thrombosis in leg veins, especially in bedridden patients. Bernhardt and colleagues discussed four cases of pulmonary embolism linked to major tranquilizers and tricyclics, highlighting these drugs’ roles in producing hyperaggregability. Singer and team reported three cases of pulmonary embolism in elderly women on major tranquilizers. This paper also presented three cases of thromboembolic events in ambulatory patients in good physical health, receiving similar medications [15].

### 3.2. Defining Antipsychotic Medications

Antipsychotic medications are broadly classified into two categories: traditional (first-generation) and novel (second-generation) antipsychotics. Traditional antipsychotics, encompassing classes such as butyrophenones (e.g., haloperidol), phenothiazines (e.g., chlorpromazine and thioridazine), and others, including indoles and thioxanthenes, were initially authorized in the 1950s, predominantly for schizophrenia management, with widespread application in a variety of psychiatric conditions [16].

In contrast, novel antipsychotics, introduced in the 1990s, comprise agents such as clozapine, risperidone, olanzapine, quetiapine, ziprasidone, aripiprazole, paliperidone, asenapine, and amisulpride. These medications exhibit considerable variability in their receptor-binding characteristics, indicating that they do not represent a uniform class of therapeutic agents. Initially sanctioned by the Food and Drug Administration exclusively for schizophrenia, these novel antipsychotics have also received approval for the treatment of bipolar mania, dementia, and large off-label practice [16].

Novel antipsychotics have increasingly been adopted as the preferred treatment option. This trend is attributed to their relative advantages over traditional antipsychotics, particularly in reducing the occurrence of extrapyramidal symptoms and tardive dyskinesia [16].

### 3.3. Defining Venous Thromboembolism

Antipsychotic medications are frequently used for managing behavioral and psychological symptoms, but they come with a range of adverse effects, impacting both the hematological and neurological systems [17]. Some of these drugs have been associated with an increased risk of venous thromboembolism, a condition that manifests primarily in two forms: pulmonary embolism and deep vein thrombosis (DVT) [18].

Pulmonary thromboembolism, often resulting from deep vein thrombosis and colloquially referred to as “economy-class syndrome”, is a noted cause of sudden death in psychiatric patients, particularly those under physical restraint. The primary risk factors for DVT, known as the Virchow triad, include decreased venous blood flow, damage to vessel walls, and enhanced blood clotting [17]. Physical restraint is thought to impede venous blood flow, while antipsychotic drugs might promote blood clotting. Venous thromboembolism represents a complex condition with multiple contributing factors [10,19]. It ranks among the top three causes of mortality related to cardiovascular disease [20,21].

Therefore, preventive measures and early diagnosis are essential to avert sudden deaths due to DVT in these scenarios [22].

The exact causes of the heightened prescription of antipsychotic medications among patients remain uncertain. However, it is suggested that this trend could be linked to an escalation in behavioral and psychological symptoms in these individuals due to the constraints enforced during the pandemic, such as limited visitor access and the suspension of group activities. Global entities such as Alzheimer’s Disease International have emphasized the augmented necessity for psychological support for those living with dementia amid the COVID-19 crisis [1].

Dispensation of antipsychotic drugs increased in the COVID-19 period compared to the pre-COVID era [1,23].

Our analysis indicated a significant association between COVID-19 and increased odds of thromboembolic events and all-cause mortality in dementia patients receiving antipsychotic medications [1].

Furthermore, it i’s theorized that individuals affected by COVID-19 might experience a disruption in coagulation balance and an intensified inflammatory response, potentially leading to a “hypercoagulable state” and elevated thrombosis risk. A study published in July 2020 indicated that antipsychotic medication treatment could aggravate respiratory issues and heighten thromboembolism risks in COVID-19 patients [24].

### 3.4. Antipsychotics and Venous Thromboembolism

The examination of data from the Food and Drug Administration Adverse Event Reporting System (FAERS) reveals a notable correlation between the use of antipsychotic medications and an escalation in thromboembolic events [25]. This link is supported by substantial statistical evidence, manifesting in significant reporting odds ratios and information components [26]. These findings bring to light a pivotal concern in medical management: the heightened risk of venous thromboembolism connected to antipsychotic drug usage [25,27].

The investigation delving into the amplified risks found links with antipsychotic medication in dementia patients, particularly under the strains of the COVID-19 pandemic. Notably, increased mortality rates in patients treated with haloperidol and risperidone, especially those infected with COVID-19, call for meticulous medication management in this sensitive group. The varying impacts of distinct antipsychotics point to the importance of strategic medication selection to minimize risks [1,28,29].

Further research, including a meta-analysis, established a clear association between the usage of antipsychotics and an augmented risk of venous thromboembolism and pulmonary embolism, with no evidence of publication bias [28,30]. Our comprehensive review of antipsychotic drug usage indicates a trend, as of 2006, toward a predominance of second-generation antipsychotics, constituting over 78% of all antipsychotic prescriptions and overshadowing the usage of first-generation antipsychotics [18].

The study uncovers a significant link between the usage of neuroleptics and increased VTE risk, with odds ratios indicating a 3.5-fold elevation in VTE risk associated with antipsychotic drugs. In contrast, antidepressant use did not show a significant correlation with VTE risk. This hospital-based case-control study reinforces the view that antipsychotic drug exposure is a potential risk factor for VTE [28,30].

In dementia patients, who often consume other medications impacting serotonin receptors and platelet function, research on the peripheral vascular effects of antipsychotics is limited. A recent nested case-control analysis within a cohort of 72,591 dementia patients indicated that current antipsychotic users in this group had a significantly higher risk of VTE compared to controls [16].

### 3.5. Contributing Factors Increasing Thromboembolic Risk

In the realm of psychiatric care, particularly in the context of antipsychotic medication use, understanding the risk of thrombosis is crucial for effective patient management. The literature provides valuable insights into various risk factors, or contributory elements, that are thought to increase the likelihood of thrombotic events in individuals prescribed these medications [18,30,31]. These factors span physiological, pharmacological, and lifestyle domains, each contributing uniquely to the overall risk profile [30]. As we delve into these factors, it is important to recognize the multifaceted nature of thrombosis risk in the context of antipsychotic therapy. The following section will explore these key risk factors, as reported in the literature, shedding light on the complex interplay of elements that may predispose patients to thrombotic complications.

### 3.6. Mostly Involved Antipsychotic Medications

Multiple research studies have pinpointed a range of acquired risk factors for venous thromboembolism [26,31]. The most commonly reported antipsychotics were quetiapine, haloperidol, olanzapine, and risperidone. Other antipsychotics were less frequently used [1,32].

Our study highlights a significant risk of pulmonary embolism in patients undergoing antipsychotic drug treatment, showing an odds ratio (OR) of 1.2, which indicates a considerable statistical significance [18]. Particularly alarming are the 30-day all-cause mortality rates in patients treated with haloperidol, and the pronounced difference in mortality rates between COVID-19 positive and negative patients receiving risperidone, suggesting a heightened risk associated with these drugs [1].

In the context of olanzapine, a case of hyperprolactinemia linked to increased pulmonary thromboembolism has been reported [33]. Interestingly, our research indicates that the elevated risk of VTE in patients treated with antipsychotics is not necessarily tied to the known risks of these medications, such as metabolic abnormalities, sedation, or hyperprolactinemia [34].

Deep vein thrombosis has been recognized as a potential complication of antipsychotic therapy, particularly with atypical antipsychotics like risperidone [35]. Additionally, numerous case reports and studies have shown an increased risk of VTE with antipsychotic use. For example, a 51-year-old female patient with bipolar disorder developed a pulmonary embolism following chlorpromazine treatment, while instances of central retinal vein occlusion have been observed in patients administered olanzapine and risperidone [36,37]. Furthermore, a case of a 39-year-old woman with chronic schizophrenia who developed acute right hemiparesis and visual field loss during a switch to clozapine therapy has been reported, leading to a provisional diagnosis of ischemic stroke [38,39].

Acute bilateral coronary artery thrombosis and myocardial infarction have also been documented in a 25-year-old man after long-term oral clozapine treatment [40]. Notably, the risk of PE varies with the type of antipsychotic used, with clozapine showing the highest associated risk (OR = 1.54). Second-generation antipsychotics, such as risperidone and ziprasidone, also present a significant risk. While previous studies have already implicated second-generation antipsychotics, such as clozapine, risperidone, and olanzapine, in PE risk, our findings additionally identify ziprasidone as carrying a significant risk for PE, a revelation that stands in contrast to other studies [18,41].

#### 3.6.1. Based on Duration of the Treatment

The duration of antipsychotic medication use is a critical factor in assessing the risk of thrombotic events, such as venous thromboembolism. This temporal correlation has been consistently observed in numerous studies, emphasizing the importance of monitoring the length of treatment when prescribing antipsychotics [35,42].

Commonly used antipsychotics, such as quetiapine, haloperidol, olanzapine, and risperidone, have been linked to an increased risk of thrombotic events, particularly with prolonged use. Our research indicates a heightened risk of pulmonary embolism in patients treated with these drugs over extended periods. This relationship points to a direct connection between the duration of antipsychotic therapy and the increased likelihood of developing thrombotic complications [18,30,42,43].

The study revealed that individuals using antipsychotic medications face a 32% higher risk of developing VTE compared to non-users. This risk escalates among current users (those with a prescription within the last three months) who experience a 56% increased risk. However, past users of antipsychotics did not show a significantly increased risk. Notably, new users of antipsychotic medications demonstrated a higher increase in VTE risk compared to those continuing with their existing medication [42].

Contrastingly, some studies found no correlation between the duration of antipsychotic drug use and the occurrence of VTE, suggesting a complex array of factors contributing to the risk [44,45]. Specifically, risperidone, a second-generation atypical antipsychotic, has been linked to cases of deep vein thrombosis, usually emerging within a period ranging from two weeks to a few months after treatment initiation [35].

Thromboembolic events have been documented within the first week of treatment with antipsychotics such as chlorpromazine and clozapine. While there are reports of risperidone-induced venous thrombosis occurring after two weeks of therapy, an extensive literature review did not reveal any cases of deep vein thrombosis specifically linked to the first week of risperidone treatment [35].

Among current users of antipsychotics, new users showed a higher risk of VTE compared to both prevalent and past users [16]. The median time frame for the diagnosis of VTE in one cohort was approximately 42 days, ranging from 16 to 94 days [46]. Our findings align with previous studies indicating that current users of antipsychotics face a greater risk of VTE than past users, suggesting that the underlying disease and the antipsychotic drugs themselves might be primary contributors to these thromboembolic events [34]. Interestingly, our study did not establish a significant dose–response relationship between the use of antipsychotic drugs and the occurrence of thromboembolic events [34].

In conclusion, the average onset time for thromboembolic conditions related to antipsychotic use was found to be about 7.49 months, with reported occurrences ranging from the third day to as late as 84 months following the initiation of treatment [20]. This underscores the need for careful monitoring of patients on antipsychotic medication, considering the varying risks associated with different durations of treatment.

#### 3.6.2. First or Second Generation

The relationship between antipsychotic medication use and the risk of venous thromboembolism and pulmonary embolism presents a nuanced picture. A key finding from one study showed no significant differences in VTE risk between first-generation antipsychotics and second-generation antipsychotics when analyzed separately, suggesting that the risk of thromboembolic events might be a general concern across different generations of antipsychotic drugs [47]. Furthermore, the study did not delve into the risks associated with individual antipsychotic drugs [16].

Contrastingly, another study highlighted a significant association between the risk of VTE and PE and exposure to second-generation antipsychotics. The risk was found to be about twice as high in individuals exposed to second-generation antipsychotics as in those not exposed. Interestingly, second-generation antipsychotics seem to have a higher likelihood of causing PE compared to VTE [30,42]. This finding underscores the need for careful consideration when prescribing second-generation antipsychotics, particularly in patients with other risk factors for thromboembolic events.

Given these findings, it is crucial for medical professionals to conduct a thorough assessment of the risk factors for each patient before initiating antipsychotic treatment. This process should involve adherence to guidelines provided by regulatory bodies and a careful evaluation of the efficacy and safety of both typical and atypical antipsychotics. Such an assessment is particularly important in the context of the patient’s specific clinical scenario, taking into account their overall health, pre-existing conditions, and potential risk factors for thromboembolic events [48].

This approach highlights the importance of individualized patient care in psychiatric treatment, emphasizing the need to weigh the benefits of antipsychotic therapy against the potential risks, especially concerning thrombotic complications.

#### 3.6.3. Antipsychotic Medication Potency

The risk of venous thromboembolism associated with antipsychotic medications appears to be influenced by the potency of the drugs, affecting both first-generation antipsychotics and second-generation antipsychotics. Notably, low-potency first-generation antipsychotics have been linked to a higher risk of VTE compared to high-potency agents [30].

A study focusing on the use of traditional antipsychotic medications found a significant association with an increased risk of idiopathic VTE compared to non-use, demonstrating an adjusted odds ratio of 7.1. This heightened risk was particularly notable with lower potency drugs such as chlorpromazine and thioridazine, which showed a stronger association with VTE (OR 24.1) compared to higher potency drugs such as haloperidol (OR 3.3). The risk was most pronounced during the initial months of treatment with conventional antipsychotic medications [49].

Additionally, our research indicates that haloperidol, classified as a high-potency first-generation antipsychotic, is associated with an increased risk of pulmonary embolism. This finding underscores the significance of a specific antipsychotic drug in determining the risk of PE [18].

Moreover, patients prescribed low-potency antipsychotic drugs were found to face a higher risk of VTE compared to those on high-potency drugs, with the odds ratio being 1.99 for low-potency drugs and 1.28 for high-potency drugs [42].

This differentiation in risk based on the potency of the antipsychotic underscores the need for careful consideration when choosing the appropriate medication, especially in patients who may be at increased risk for thromboembolic events.

### 3.7. Antiphospholipid Antibodies

Furthermore, the use of some psychotropic drugs, including chlorpromazine and clozapine, correlates with increased levels of anti-phospholipid antibodies (aPLs), which are thrombogenic. Interestingly, increased aPL levels can also be a primary condition in schizophrenic patients. Research by Canoso et al. found a significantly higher prevalence of autoantibodies, such as antinuclear antibodies, aPLs, rheumatoid factor, and immunoglobulin M, in chronic psychiatric patients on long-term neuroleptic therapy compared to normal controls [44].

In contrast to observations in systemic lupus erythematosus and similar autoimmune conditions, chlorpromazine does not seem to correlate with a heightened incidence of thrombosis [50].

### 3.8. Endothelial Involvement

Endothelial dysfunction has emerged as a significant concern linked to the use of antipsychotic agents [45]. Atypical antipsychotics, in particular, have been implicated in elevating the risk of vascular dysfunctions, which, in turn, are associated with an increased susceptibility to cardiovascular diseases [21]. The intricate relationship between antipsychotic medications and endothelial health warrants an in-depth exploration of the multiple facets contributing to this connection [51,52].

Emerging research suggests that various drugs, including antipsychotic agents, have the capacity to impede the vasoprotective mechanisms maintained by the endothelium. By tampering with these mechanisms, these medications can potentially pave the way for the development of cardiovascular diseases. The specific pathways and molecular interactions through which antipsychotic agents influence endothelial function remain subjects of ongoing investigation [53,54].

Curiously, a study conducted on schizophrenia patients undergoing antipsychotic drug therapy explored the interplay between genetic variants and metabolic syndrome concerning endothelial function. The investigation unveiled noteworthy associations between genetic variants of endothelial nitric oxide synthetase and endothelial dysfunction [55]. This connection provides a critical link between the genetic makeup of patients and their response to antipsychotic treatment, shedding light on individualized approaches to care [56].

In the quest to understand the dynamics of endothelial function among somatically healthy schizophrenia patients treated with atypical antipsychotic agents, researchers made a significant discovery, revealing elevated levels of asymmetric dimethylarginine (ADMA), an endogenous inhibitor of nitric oxide synthase. Elevated ADMA levels serve as pertinent markers of endothelial dysfunction, emphasizing the elaborate cellular-level interactions influenced by antipsychotic medications [57].

An additional dimension of this multifaceted relationship is the observation that plasma levels of vascular endothelial growth factor (VEGF) exhibit variation in patients with schizophrenia, particularly prior to antipsychotic treatment. Research findings indicate that VEGF levels are lower in these patients before the commencement of treatment. However, a compelling shift occurs after the administration of antipsychotic agents, with VEGF levels showing a subsequent increase. This dynamic points to a complex interplay between antipsychotic treatment and the intricate mechanisms involved in endothelial health [58,59].

### 3.9. Platelet Aggregation

Recent investigations have uncovered an intriguing association between the use of clozapine and the occurrence of thrombosis. While clozapine’s direct interaction with fibrinogen does not compromise its structural integrity, the drug markedly influences fibrin formation. Specifically, clozapine slows down the coagulation process and results in thinner fibrin fibers. This phenomenon suggests that clozapine may confer thrombogenic properties to fibrinogen, potentially in a dose-dependent manner. Consequently, the dosage of clozapine could be a pivotal factor in determining its influence on fibrinogen and the overall coagulation process within the body [45,60].

Contrary to the hypothesis that increased platelet aggregation due to activation of serotonin receptor 2A (5-HT2A) leads to venous thromboembolism, some findings do not support this theory. Aripiprazole and quetiapine, both of which act on 5-HT2A receptors, have not shown a significant increase in the risk of pulmonary embolism. Furthermore, in vitro studies have failed to demonstrate an increase in platelet aggregation with the use of antipsychotics, such as haloperidol, olanzapine, or risperidone. This indicates that the link between antipsychotic drug use and PE risk is multifaceted and cannot be solely attributed to the sedative effects or serotonin receptor activation [18].

Prolactin and leptin, hormones known for their involvement in various physiological processes, have been identified as significant coactivators in adenosine diphosphate (ADP)-dependent platelet aggregation and P-selectin expression. These findings suggest their potential role as risk factors in both arterial and venous thrombosis. Clinical conditions that typically elevate prolactin or leptin levels, such as pregnancy, obesity, or treatment with antipsychotic drugs, have also been associated with an increased risk of thromboembolic events [61]. This correlation underscores the importance of considering hormonal influences, particularly prolactin and leptin, in the context of thrombosis risk when using antipsychotic medications [61].

In summary, the role of platelets in thrombosis associated with antipsychotic drug use is complex and involves multiple pathways and factors. The impact of clozapine on fibrin formation, the lack of a direct link between 5-HT2A receptor activation and increased PE risk, and the potential involvement of hormones like prolactin and leptin in platelet aggregation all contribute to our understanding of thrombosis in the context of antipsychotic medication use [60,61]. These insights are crucial for guiding clinical decisions and risk assessments in patients undergoing antipsychotic therapy.

### 3.10. Immobilization

The hypothesis that sedation induced by antipsychotic drugs contributes to an increased risk of pulmonary embolism is a subject of ongoing research and debate. While all antipsychotics potentially have sedative effects, the intensity of these effects varies depending on the specific drug [62]. For example, quetiapine is known for its sedative properties, yet it does not seem to be associated with an increased risk of PE [63]. Interestingly, the sedative effect of quetiapine may diminish over time. In contrast, ziprasidone, which is not typically linked to sedation, has been found to show a significant risk of PE [18].

Sedation induced by certain antipsychotic drugs, such as chlorpromazine, clozapine, olanzapine, and quetiapine, can reduce patient movement, potentially contributing to blood stasis [64]. Blood stasis is a well-recognized risk factor for venous thromboembolism [44]. This sedative effect, therefore, could be a contributing factor to the increased risk of thrombotic events in patients treated with these medications [27].

In studies of venous thromboembolism, risk factors present in at least 15% of both studied cohorts included acute infection or rheumatologic disorder, obesity, and the use of antipsychotic medication. Notably, in one of the cohorts, the two most prevalent risk factors were the use of an antipsychotic agent (observed in 100% of cases) and reduced mobility [46]. This suggests a strong association between antipsychotic use, reduced mobility, and the risk of VTE [62,65].

Furthermore, the study highlights a significant link between prolonged physical immobilization and the risk of venous thromboembolism in psychiatric patients, especially those receiving antipsychotic therapy [63,66]. Autopsy findings in five patients where pulmonary thromboembolism was identified as the direct cause of death emphasize the critical need to monitor and manage periods of immobilization in these patients to mitigate the risk of VTE [67,68]. This underlines the importance of physical activity and mobility in patients undergoing antipsychotic treatment as preventive measures against thrombotic risks.

### 3.11. Obesity

The relationship between antipsychotic drug use and obesity is particularly noteworthy, as obesity itself is a known risk factor for deep vein thrombosis [46]. Antipsychotic agents, such as clozapine and olanzapine, are frequently associated with an increased risk of obesity. It was observed that obese individuals had higher levels of blood clotting factors VIII and IX compared to controls. This elevation in clotting factors among obese patients underscores the augmented risk of thrombosis in this population [44].

In a specific cohort, the presence of a body mass index (BMI) of 30 kg/m^2^ or higher was noted in 30% of the participants. This prevalence of obesity within the cohort points to its significant role as a risk factor for venous thromboembolism [46]. The high incidence of obesity in this group further emphasizes the importance of considering body weight and related metabolic factors when assessing the risk of VTE [62].

Moreover, in individuals diagnosed with schizophrenia or bipolar disorder, the use of antipsychotic medications, such as olanzapine and clozapine, has been linked to various metabolic irregularities [69]. These include substantial weight gain, disruptions in lipid profiles, and alterations in glucose metabolism [16,21].

In this scenario, a case of branch retinal vein occlusion was reported in which a young patient, initially with normal lab results, experienced dyslipidemia after two years of quetiapine use. Given that such antipsychotics can lead to thrombotic episodes through metabolic imbalances, it is essential to monitor and address any signs of dyslipidemia and obesity promptly to prevent thrombotic complications [70].

### 3.12. Age

Age-related variations in the risk of venous thromboembolism in the context of antipsychotic and antidepressant drug use present a complex clinical picture. While some studies suggest a heightened risk, others indicate a more nuanced scenario [71]. For instance, research involving adults aged 65 and older found no significant increase in VTE risk associated with the use of these medications [72]. This finding challenges the commonly held perception of a universally elevated thromboembolic risk with antipsychotic drug use in older adults [73].

In a detailed study of the elderly population encompassing 111,818 patients, no substantial correlation was found between the current use of antipsychotics and the incidence of VTE. This indicates that the thromboembolic risk profile in the elderly may differ from that in other age groups, suggesting a distinct response to these medications in older patients. This study considered various factors, such as medication dosage and duration, yet still reported no increased risk [71].

Certain antipsychotic agents, particularly depot preparations of thioxanthenes such as zuclopenthixol and flupenthixol, have been linked to rare instances of hypocoagulability. This condition arises due to the development of autoantibodies against factor VIII, a critical component in the coagulation cascade. These antibodies, primarily of the immunoglobulin G4 subclass, inhibit factor VIII, leading to reduced clotting capability. This immune response, resulting in acquired hemophilia A, is a rare but significant hematological side effect of the prolonged use of these antipsychotics [74]. However, case reports indicate atypical occurrences where the expected clinical risk profiles for zuclopenthixol use are contradicted, suggesting a more intricate interplay of underlying mechanisms [75].

An estimation from a study suggested that there are an additional 4 cases of VTE per 10,000 patients treated annually across all ages, with the number rising to 10 per 10,000 in patients aged 65 and over. Interestingly, younger patients are found to have an approximately threefold higher risk of PE and VTE compared to older patients [72].

However, case reports provide evidence of exceptions where typical risk factors are absent, contradicting typical clinical expectations and risk profiles for the use of zuclopenthixol and indicating a more complex interplay of the underlying mechanisms [75].

In the context of COVID-19, mortality is influenced by several factors, including gender, and the presence of cardiovascular and metabolic comorbidities, such as diabetes, obesity, chronic renal failure, and chronic heart disease. Medications such as antipsychotics, antidepressants, and antiepileptics were found to be significantly associated with increased COVID-19 mortality in both the Aragon and Campania regions. This highlights the importance of considering chronic baseline treatments for conditions that predispose patients to systemic inflammation and thrombosis in managing COVID-19 patients [1,2].

Furthermore, a correlation was observed between the use of antipsychotic drugs and an increased occurrence of thrombotic events, especially in the very elderly [76]. Additionally, individuals over the age of 40 are recognized as having an elevated risk of VTE, with this risk doubling with each passing decade. This information underscores the necessity of a nuanced approach to assessing and managing thromboembolic risks associated with psychiatric medications, especially considering age-related factors [1,2].

It is commonly recognized as a primary determinant in the mortality associated with infections. Additional factors contributing to this include being male and the presence of cardiovascular and metabolic comorbidities, such as diabetes, obesity, chronic renal failure, and chronic heart disease. Central to both these conditions and the pathophysiology of COVID-19 is their impact on the body’s inflammatory response and the functioning of both the immune and coagulation systems [2].

In Table 1, we summarized the main findings.

## 4. Discussion

### 4.1. COVID-19 and Thrombosis

Thrombosis, a serious concern in COVID-19, affects approximately one-third of patients, most severely leading to pulmonary embolism [77,78]. The development of thrombosis is influenced by multiple interacting factors, commonly known as Virchow’s triad, including vascular endothelial damage, venous stasis, and hypercoagulability [79]. There seems to be an underlying mechanism causing a response severe enough to still cause venous thromboemboli in prophylactically anticoagulated patients [80]. This, plus the thrombotic and microangiopathic thrombotic findings on COVID-19 patients’ autopsies, confirms the importance of this topic [81].

Basically, all coagulation parameters have been shown to be potentially altered in COVID-19 infection, but not all of them show clear correlation with the extent of the disease process [82].

While immobilization in severe COVID-19 cases surely plays a role in venous stasis, other effects are not as straight forward and are yet to be explored in detail [82].

SARS-CoV2 can enter the body in a variety of ways but infects through the binding of its S-protein to the angiotensin converting enzyme 2 (ACE2) receptor. ACE2 can be found in a variety of organs, including the nose, bronchi, blood vessels, heart, kidney, and brain [83]. Physiologically, ACE2 converts angiotensin II to angiotensin 1-7. Since it is being used by the virus, it has been hypothesized that less ACE2 is available for normal bodily function, leading to an increase in angiotensin II and a decrease in angiotensin 1-7. Angiotensin II causes vasoconstriction, which can lead to capillary congestion, with microthrombi in the alveolar capillaries [79]. In addition, ATII is a potent pro-inflammatory peptide hormone that causes the accumulation of reactive oxygen species (ROS) through NAD(P)H oxidase. Reactive oxygen species, in turn, are known to play a role in vascular inflammation and contribute to endothelial dysfunction [84], while Angiotensin 1-7 actually has anti-inflammatory and anti-thrombotic effects [85]. This dysregulation of the Renin-Angiotensin-Aldosterone-System causes endothelial dysfunction not only by ROS, but also by overexpressing various factors and receptors, such as COX-2, VEGF, and LOX-1 [83]. Another potential culprit involved is von Willebrand factor. Physiologically, von Willebrand factor is found sub endothelially, where it is released through endothelial damage, aiding platelet aggregation and ultimately thrombosis. A single-center, cross-sectional study from Yale–New Haven Hospital found a greatly increased amount of von Willebrand factor in COVID-19 patients. This amount was correlated with disease severity (ICU vs. non-ICU patients) [86].

It was discovered fairly early that a major part of COVID-19′s pathological effects are caused by an excessive immune response, including but not limited to the complement system, neutrophil extracellular traps (NETs), and mitogen-activated protein kinases (MAPKs) pathways. Commonly implicated is the release of inflammatory cytokines, namely IL-1, IL-6, IL-8, and IL-17. Cytokine IL-6 plays a vital role in hypercoagulability by magnifying fibrinogen and platelet production and is positively correlated with COVID-19 severity [87]. The “cytokine storm” is not necessarily found system-wide, but can also be triggered locally in the lung, causing local thrombus formation. This will lead to the common picture of pulmonary emboli without DVTs, as we encounter it in COVID-19 [88]. While cytokines, von Willebrand factor, and fibrinogen are responsible for increased thrombotic factors, they also cause a state of hypercoagulability due to the increased number of plasma components [79].

In addition to the triggered immune response, hypoxia, which is commonly encountered in COVID-19 patients, also stimulates thrombosis. Hypoxia causes expression of hypoxia-inducible transcription factors, causing activation of thrombosis-related genes [82].

There are specific genetic factors that can elevate the risk of thrombosis. However, research exploring the connection between genetic mutations and COVID-19-related thrombosis produces conflicting results, necessitating further investigation [79,86,88].

### 4.2. Limitations and Future Research Directions

While our study provides important insights, it also has limitations. The observational nature of the study restricts our ability to establish causality. Some studies suggest that patients with schizophrenia experience a higher incidence of venous thromboembolism compared to the general population [31].

Future research should focus on longitudinal studies to better understand the long-term impacts of antipsychotic use in patients, especially in the context of pandemic-related stressors and restrictions. Additionally, more research is needed to explore the mechanisms behind the varying impacts of different antipsychotic medications in the context of COVID-19 [1,71,73].

### 4.3. Need for Cautious Medication Management

Given the heightened risk of adverse outcomes, particularly thromboembolic events and mortality, healthcare providers should exercise heightened caution when prescribing antipsychotics for patients during pandemic conditions like COVID-19. Alternatives to antipsychotics or strategies to minimize exposure may be beneficial, especially for patients with additional risk factors for severe COVID-19 outcomes [1,23].

Evidence indicates that complications arising from the use of antipsychotic drugs are not only common, but also incur substantial costs. This situation underscores the necessity for the introduction of an effective algorithm in clinical practice, particularly in psychiatry, to mitigate these complications. Implementing protocols is crucial to reducing both the frequency of these adverse events and the associated healthcare expenses [46].

New risk prediction algorithms are now incorporating the use of antipsychotics as specific predictor variables in assessing the risk of venous thromboembolism. These algorithms are designed to more accurately evaluate the potential of antipsychotics to induce VTE, reflecting the nuanced risks associated with the different types of these medications. This approach marks a significant advancement in personalized medical assessments, particularly for patients undergoing antipsychotic therapy [89,90].

### 4.4. Our Contribution

This study contributes to the growing body of evidence concerning the adverse health outcomes linked to some of the antipsychotic drugs. Recent research has already established the significantly heightened risks of severe events and mortality in patients treated with antipsychotics for behavioral issues [42]. However, our findings warrant further validation through replication in another database before any modifications in clinical practices are suggested. To accurately assess the risks associated with specific antipsychotics, larger datasets are necessary.

## 5. Conclusions

Subsequent studies should corroborate our results, thus advocating for a more cautious approach in prescribing antipsychotic drugs, particularly for conditions like nausea and agitation, and especially in patients with a high risk of thromboembolism. Patients should be well informed about the risk–benefit balance of these drugs prior to starting treatment.

Achieving this requires the development of new algorithms capable of estimating an individual’s absolute risk of thromboembolism. These algorithms should incorporate individual-level factors, such as age, sex, socioeconomic status, smoking habits, comorbidities, and concurrent medication use. Such a tailored approach would enhance patient care by providing a more nuanced understanding of the risks associated with antipsychotic drug therapy and enabl us to select the best individualized treatment.

## Figures and Tables

**Table 1:** Risk factor analysis table for VTE in patients on antipsychotic medication.

Risk Factor	Description	Associated Antipsychotics	Impact on VTE Risk
Prolonged Immobilization	Reduced mobility due to sedative effects of drugs	Various antipsychotics	Immobility increases the risk of blood stasis, leading to thrombosis
Hypercoagulability	Altered coagulation pathways	Various antipsychotics	Certain antipsychotics may promote blood clotting, enhancing thrombosis risk
Endothelial Dysfunction	Impairment of endothelial function	Atypical antipsychotics	Endothelial dysfunction can contribute to cardiovascular diseases
Genetic Predispositions	Interaction with genetic factors	Various antipsychotics	Genetic factors can interact with medications to heighten VTE risk
COVID-19 Infection	Exacerbation of VTE risk due to COVID-19	Various antipsychotics	COVID-19 may disrupt coagulation balance, increasing thrombosis risk
Age and Comorbidities	Higher risk in older patients and those with comorbidities	Various antipsychotics	Older age and comorbidities like obesity increase VTE risk
Specific Medication Types	Different risks associated with specific drugs	Clozapine, Risperidone	Some antipsychotics, like clozapine, have a higher associated risk of PE

## Data Availability

Data available upon request.
